# Supplementary material for: Efficacy of acupuncture on lower limb motor dysfunction following stroke: A systematic review and meta-analysis of randomized controlled trials
Source: PLoS One. 2025 May 7;20(5):e0312918. doi: 10.1371/journal.pone.0312918 (PMC12057971; doi:10.1371/journal.pone.0312918)
Supplement: Attachment — Submitted filename: Response to Reviewers 2.docx [file pone.0312918.s002.docx]

Dear Editor,

Thank you for your request regarding data availability. In compliance with PLOS ONE's data sharing policy, I have prepared the complete dataset used in our meta-analysis as supplementary files. These files contain detailed search strategies and extracted data for meta-analysis to ensure full replication of our findings.

The following data files have been uploaded as supporting information along with the manuscript submission:

Search_Strategy.docx: Comprehensive literature search strategies, including all databases searched, combination of search terms, and search dates

Data_Extraction.xlsx: Raw extracted data containing:

Basic information of included studies (authors, publication year, country, etc.)

Study characteristics

Effect size data

Quality assessment data

PRISMA_Flow.pdf: Literature screening flow diagram

All files have been systematically organized with clear labels and descriptions to ensure that other researchers can understand and replicate our analytical process.

Please do not hesitate to contact me if any additional information or alternative format is required for the data sharing requirement.

Best regards.
