# Supplementary material for: Efficacy of acupuncture on lower limb motor dysfunction following stroke: A systematic review and meta-analysis of randomized controlled trials
Source: PLoS One. 2025 May 7;20(5):e0312918. doi: 10.1371/journal.pone.0312918 (PMC12057971; doi:10.1371/journal.pone.0312918)
Supplement: Attachment — Submitted filename: Response to Reviewers.docx [file pone.0312918.s003.docx]

Response to Reviewers

Dear Editor,

We thank you and the reviewers for your careful evaluation of our manuscript. We have addressed all the comments and made the necessary revisions to improve our paper. Below, we provide point-by-point responses to each of the concerns raised.

Response to Editor's Comments:

We have carefully reviewed our reference list and confirmed that all citations are complete, accurate, and up-to-date. No retracted articles were cited in our manuscript.

Response to Reviewer #2:

We thank the reviewers for their positive feedback on our previous revisions. As suggested, we have expanded the introductory section to better explain the physiological mechanisms of acupuncture. Specifically, we have added: 1. an overview of the known neurophysiological pathways that are activated during acupuncture stimulation, including the role of the peripheral and central nervous systems. 2. A discussion of the mechanisms by which acupuncture improves motor dysfunction after stroke.

We believe these revisions have enhanced the scientific rigor of our study. We welcome any additional suggestions for further improvements.
